# Multiple Fingerprint–Activity Relationship Assessment of Immunomodulatory Polysaccharides from *Ganoderma lucidum* Based on Chemometric Methods

**DOI:** 10.3390/molecules28072913

**Published:** 2023-03-24

**Authors:** Jing Liu, Jingsong Zhang, Jie Feng, Chuanhong Tang, Mengqiu Yan, Shuai Zhou, Wanchao Chen, Wenhan Wang, Yanfang Liu

**Affiliations:** 1 Institute of Edible Fungi, Shanghai Academy of Agricultural Sciences, Key Laboratory of Edible Fungi Resources and Utilization (South), Ministry of Agriculture, National Engineering Research Center of Edible Fungi, Shanghai 201403, China; lj15735649029@163.com (J.L.); syja16@saas.sh.cn (J.Z.); fengjie@saas.sh.cn (J.F.); tangchuanhong@saas.sh.cn (C.T.); yanmengqiu@saas.sh.cn (M.Y.); zhoushuai@saas.sh.cn (S.Z.); chenwanchao@saas.sh.cn (W.C.); wenhanwang@saas.sh.cn (W.W.); 2 Key Laboratory of Synthetic and Biological Colloids, Ministry of Education, School of Chemical and Material Engineering, Jiangnan University, Wuxi 214122, China

**Keywords:** *Ganoderma lucidum* polysaccharides, immune activity, regression analysis, fingerprint–activity relationship

## Abstract

Polysaccharides with molecular weights ranging from 1.75 × 10^3^ to 1.14 × 10^4^ g/mol were obtained from the fruit bodies of *Ganoderma lucidum*. The multiple fingerprints and macrophage immunostimulatory activity of these fractions were analyzed as well as the fingerprint–activity relationship. The correlation analysis of molecular weight and immune activity demonstrated that polysaccharides with molecular weights of 4.27 × 10^3^~5.27 × 10^3^ and 1 × 10^4^~1.14 × 10^4^ g/mol were the main active fractions. Moreover, the results showed that galactose, mannose, and glucuronic acid were positively related to immunostimulatory activity. Additionally, partial least-squares regression and grey correlation degree analyses indicated that three peaks (P2, P3, P8) in the oligosaccharide fragment fingerprint significantly affected the immune activity of the polysaccharides. Hence, these ingredients associated with activity could be considered as markers to assess *Ganoderma lucidum* polysaccharides and their related products, and the study also provides a reference for research on the spectrum–effect relationship of polysaccharides in the future.

## 1. Introduction

*Ganoderma*, known as “Lingzhi,” “miraculous zhi,” or “auspicious herb,” has been used as a health-improving medicine in China for more than 2000 years [1] and is traditionally considered as the symbol of good fortune and longevity. In early Chinese history, the Shen Nong’s Herbal Classic, one of the most influential Chinese traditional medicine books, recorded that *Ganoderma* could enhance immunity, calm the mind, and relieve coughing [2]. There are about 98 species of *Ganoderma* in China, but only the dried fruit bodies of *Ganoderma lucidum* (*G*. *lucidum*) and *Ganoderma sinense* (*G*. *sinense*) are recorded in the Chinese Pharmacopoeia. Since *G*. *lucidum* and *G*. *sinense* were officially permitted for use in healthy foods according to the prescript of the Chinese Food and Drug Administration, they have become a research hotspot. Moreover, *G*. *lucidum* has been used for hundreds of years due to its remarkable biological activity, such as in the treatment of cancer [3]. *Ganoderma lucidum* contains complex components, including polysaccharides, triterpenes, sterols, and nucleosides [4,5]. Among these components, *Ganoderma lucidum* polysaccharides have received extensive attention due to their biological activities, such as immune regulation [6], anti-tumor [7], liver protection [8], and hypoglycemic effects [9]. Until now, more than 200 kinds of polysaccharides have been extracted and isolated from *Ganoderma* [10]. Most polysaccharides with molecular weights ranging from thousands to millions are comprised of glucose, mannose, galactose, fucose, and arabinose with different combinations and types of glycosidic linkages [11]. In addition, the structural features of these polysaccharides, including molecular weight, monosaccharide composition, and glycosidic linkages, affect their biological activities [12,13,14]. As complex biomacromolecules, it is hard to evaluate the quality of polysaccharides and their related products.

Fingerprint technology is an effective method used to characterize complicated mixture systems of multiple chemical compounds [15], and it has been used for the qualitative identification of polysaccharides from medicinal plants as well as edible fungi in recent years. The fingerprints of molecular weight [16], monosaccharide composition [17], and partial hydrolysis products [18] of polysaccharides are established by chromatography and analyzed further with chemometric methods, which can be applied to distinguish raw materials. For instance, Liu et al. analyzed the monosaccharide composition fingerprints of polysaccharides from *Poria cocos* (*P. cocos*) and *Polyporus umbellatus* (*P. umbellatus*) [19], respectively, and found that the monosaccharide types of *P. umbellatus* were more complex than those of *P. cocos*. Meanwhile, *P. cocos* and *P. umbellatus* polysaccharides could be divided into two classifications by principal component analysis (PCA), which indicated that ribose and mannose were the main monosaccharides distinguishing *P. cocos* and *P. umbellatus*. It was also reported that the molecular weight profiles of crude polysaccharides from *G. lucidum* and *G. sinense* exhibited two similar peaks, and some samples of *G. lucidum* could be hydrolyzed by cellulase and β-mannanase whereas all samples of *G. sinense* showed negative responses to enzymatic hydrolysis [20]. These studies were all focused on the fingerprints of crude polysaccharides and the results revealed the diversity of polysaccharides in terms of monosaccharide composition and glycosidic linkages. Indeed, ignoring differences in the hydrolysis conditions of different polysaccharide fractions, the crude polysaccharide fingerprint obtained under specific hydrolysis conditions could not accurately reflect the features of the polysaccharides.

Previous studies proved that polysaccharides with molecular weights (*M_w_*) ranging from 10^3^ to 10^6^ g/mol in *G. lucidum* were mainly composed of α/β-glucans and heteropolysaccharides [11]. β-Glucans with high *M_w_* have been purified from the fruit bodies, spores, and mycelia of *Ganoderma lucidum*, which are considered as a biological response modifier and they possess significant immune and antitumor activities [21,22]. However, β-glucan could not be hydrolyzed efficiently due to its special structure, implying that the hydrolysis conditions of β-glucan are different from those of other polysaccharides. Hence, it is necessary to further isolate the crude polysaccharides and explore the characterization of different fractions using appropriate hydrolysis conditions. Meanwhile, fractions with low *M_w_* were the main components of *G. lucidum* polysaccharides, and some studies have reported their structures and diverse biological activities [23,24]. However, the relationship between the characteristics and activities of these polysaccharides remains unclear, especially the spectrum–effect relationship. Thus, the main components and features of the polysaccharides contributing to bioactivity need to be further investigated based on the fingerprint–activity model, which is helpful for quality control of polysaccharides in raw materials.

In this study, the molecular weight distribution, monosaccharide composition, and partial acid hydrolysates fingerprints of *G. lucidum* polysaccharides with low *M_w_* were constructed and analyzed, and the immunological activities of these polysaccharides were evaluated in vitro. In addition, the relationship between immune activity and the polysaccharide fingerprint was investigated by grey relational analysis combined with linear regression analysis. Characteristic peaks related to the immune activity of *G. lucidum* polysaccharides were identified through the fingerprint–activity model, which provides a scientific reference for the quality evaluation of polysaccharides and their related products and also lays a foundation for research on the profile–effect relationship of polysaccharides.

## 2. Results and Discussion

### 2.1. Yield and Sugar Content of Polysaccharides

Water extraction and precipitation with ethanol are commonly used to isolate polysaccharides based on their solubility [25,26]. This method for separation is time-saving and easily prepares sufficient quantities of polysaccharides for subsequent investigation. Polysaccharides were isolated from *Ganoderma lucidum* fruit bodies through hot water extraction followed by ethanol precipitation in our study. The yields were 0.18%~0.74% (in Table 1) based on the weight of the fruit bodies. The polysaccharide content of these samples ranged from 39.59% to 48.96%. Moreover, previous work indicated that polysaccharides with *M_w_* of 10^4^ g/mol were the main fraction of crude polysaccharides from *Ganoderma lucidum* [20]. In general, there was a slight difference in the polysaccharide content of these samples. Thus, it was difficult to evaluate and identify the polysaccharides in light of the sugar content. To study the fractions with low *M_w_* in detail, the functional groups, molecular weight distribution, monosaccharide composition, and oligosaccharide fragments of the polysaccharides were further investigated.

### 2.2. FTIR Analysis

**Figure 1:**
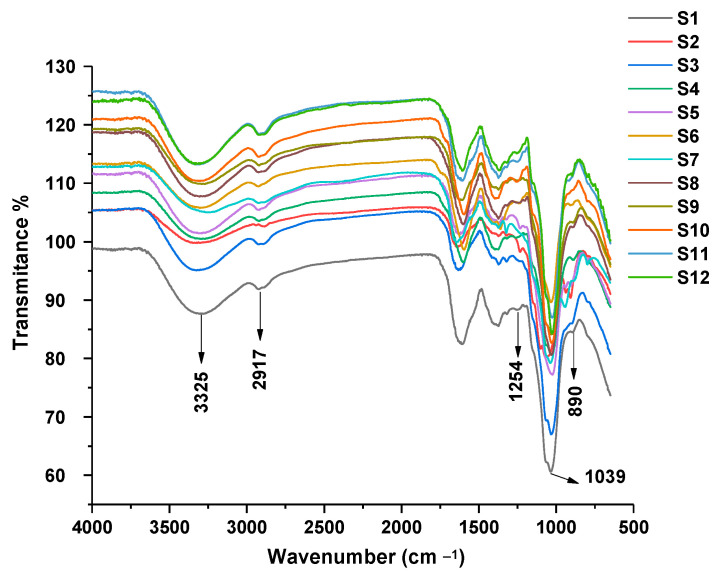
FTIR spectra of *Ganoderma lucidum* polysaccharides.

FTIR is mainly used to analyze the functional groups of polysaccharides to prove the existence of polysaccharide fractions, and some signals can be used to distinguish the anomeric carbon configuration of polysaccharides. The FTIR spectra of *Ganoderma lucidum* polysaccharides are depicted in Figure 1. There was one prominent band near 3325 cm^−1^ that represented the stretching vibration of O-H, and a signal at 1254 cm^−1^ corresponded to the variable angle vibration of O-H. The characteristic band at 1039 cm^−1^ corresponded to the asymmetrical stretching vibration of C-O-C. The absorption band at 2917 cm^−1^ was identified, which was attributed to the stretching vibration from C-H. Furthermore, the absorbance of a typical band at 890 cm^−1^ in the spectra was assigned to the characteristic configuration of β glycosides [27]. The results showed that the samples all had the typical characteristic bands of polysaccharides and the spectral similarity of different polysaccharides was high.

### 2.3. Fingerprint Characterization Analysis

#### 2.3.1. Molecular Weight Mapping Based on HPSEC

**Figure 2:**
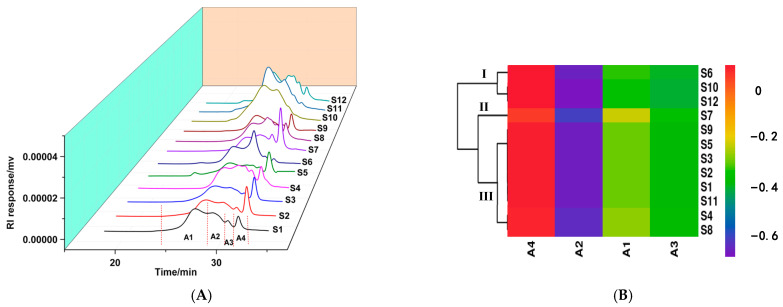
Molecular weight distribution of *Ganoderma lucidum* polysaccharides. HPSEC fingerprint (**A**), hierarchical cluster analysis of HPSEC fingerprint (**B**).

Molecular weight is an important structural feature closely related to the activity of polysaccharides, which can be used for quality control of herbal material and its products [28]. The molecular weights of polysaccharides were determined using HPSEC equipped with multiple detectors. As shown in Figure 2A, there were four chromatographic elution peaks in the HPSEC profiles of the polysaccharides from different samples. However, the proportions of each peak were significantly different, and peaks A1 and A2 were the main components in the polysaccharides based on the peak area percentages. The molecular weights of peaks A1, A2, A3, and A4 were in the range of 1 × 10^4^~1.14 × 10^4^, 4.27 × 10^3^~5.27 × 10^3^, 2.60 × 10^3^~2.81 × 10^3^, and 1.75 × 10^3^~1.86 × 10^3^ g/mol, respectively. Some research has indicated that the molecular weights of polysaccharides affect their bioactivities. For instance, two polysaccharides with different *M_w_* were obtained from *Ganoderma lucidum*, and GLP-1 with a larger *M_w_* (1 × 10^5^ g/mol) showed better immunomodulatory activity by increasing the weights of immune organs [29]. Other researchers reported that fractions with low *M_w_* (*M_w_* < 1.2 × 10^4^ g/mol) could stimulate macrophages to secrete more cytokines [30]. These results suggested that the bioactivities of polysaccharides are closely associated with *M_w_*, and more effort needed to be put into studies regarding the relationship between *M_w_* and activity. Therefore, the association between *M_w_* and immune activity of the fractions was evaluated in the current study.

Clustering analysis (HCA), also called group analysis, is based on the fundamental principle of “like attracts like” [31]. It clusters research objects with similar attributes into one category, which means that samples with higher similarity will be clustered into one category. The normalized data of the common peak areas of the fingerprint formed the data matrix. Then, the data matrix was imported into SPSS 26.0 Statistics software for HCA.

As shown in Figure 2B, the samples were divided into three categories based on their molecular weight distribution. S6, S10, and S12 were clustered into category I, which contained high proportions of A1 (62.48%~41.72%) and A2 (48.18%~26.41%). Category II only contained S7, with the highest proportion of A4 fraction (29.7%), while category III contained 8 batches of polysaccharides, most of which possessed relatively high proportions of A4. Samples from different sources clustered into one group, which indicated that the molecular weight distribution of fractions from the above sources was very similar.

#### 2.3.2. Monosaccharide Composition Mapping Based on HPAEC

**Figure 3:**
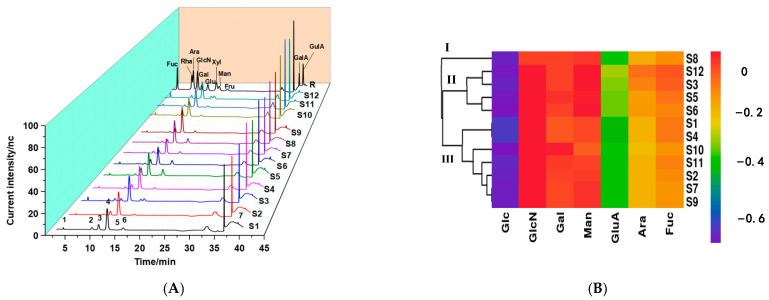
Monosaccharide composition of *Ganoderma lucidum* polysaccharides. HPAEC fingerprint (**A**), hierarchical cluster analysis of HPAEC fingerprint (**B**). 1: fucose, 2: arabinose, 3: glucosamine, 4: galactose, 5: glucose, 6: mannose, 7: glucuronic acid, R: standards profile.

Monosaccharides are the basic units of polysaccharides, and the complex structures of polysaccharides are due to differences in the type, connection order, and glycosidic linkages of monosaccharides. Additionally, monosaccharide composition is an important primary structural feature of polysaccharides, which is closely related to their physicochemical properties and biological activities [32,33]. The monosaccharide composition of the samples was determined by HPAEC. As shown in Figure 3A, seven common peaks (from 1 to 7) were detected in the hydrolysates of all samples, which were identified as fucose (Fuc), arabinose (Ara), glucosamine (GlcN), galactose (Gal), glucose (Glc), mannose (Man), and glucuronic acid (GluA) according to the retention times of the monosaccharide standards. It was observed that Glc (54.16%~85.76%) was the main monosaccharide (in Table 2), followed by Gal (4.88%~39.40%) and Man (0.26%~20.51%), with a small amount of Fuc, GlcN, GluA, and Ara. The results were consistent with another report that *Ganoderma lucidum* polysaccharides were mainly composed of Glc, Man, and Gal [24], but there were some differences due to several factors, such as the sources of the *Ganoderma lucidum* fruits and the extraction and purification methods of the polysaccharides [34,35].

The HCA results for monosaccharide composition are presented in Figure 3B. The hierarchical cluster diagram was more complex than that for molecular weight and indicated that differences in monosaccharide composition among samples were more obvious. The polysaccharides from different samples could be classified into three categories. S8, with a higher proportion of Man and Glc, was clustered into category I. Category II included four batches of polysaccharides (S6, S3, S5, and S12), which had similar monosaccharide compositions. The remaining seven samples were clustered into category III, among which S10 and S11 contained the highest Gal proportion and lowest Man proportion. Moreover, the results indicated that the monosaccharide compositions of the polysaccharides were diverse, although the molecular weight distributions were very similar.

#### 2.3.3. Partial Acid Hydrolysate Mapping Based on PMP–HPLC

**Figure 4:**
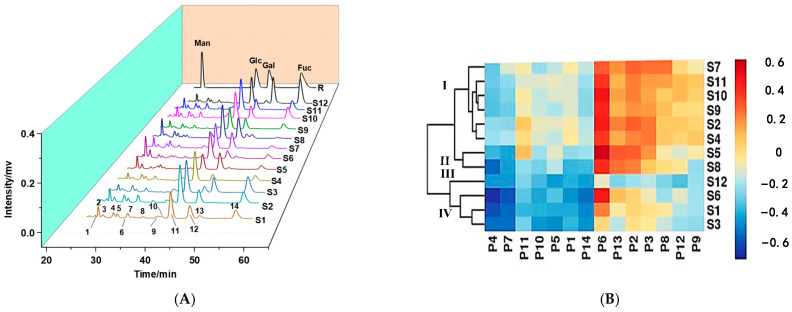
Partial acid hydrolysates of *Ganoderma lucidum* polysaccharides. PMP–HPLC fingerprint (**A**), hierarchical cluster analysis of PMP–HPLC fingerprint (**B**). R: standards profile.

Oligosaccharides, the degradation products of polysaccharides, can reflect some structural characteristics of polysaccharides. Moreover, researchers found that oligosaccharides with different structures showed differences in physiological activities, which indicated that oligosaccharides play an important role in the activity of polysaccharides [36]. To further characterize the structural information of the polysaccharides, partial acid hydrolysis was used to prepare the hydrolysate fragments containing oligosaccharides and PMP–HPLC was used for the separation and detection of the hydrolysates. As shown in Figure 4A, 14 common peaks were observed in the HPLC fingerprint analysis results, indicating that the components in the hydrolysates were complex and diverse. According to the proportions of peak areas (in Table S2), eight peaks including P2 (3.1%~15.36%), P3 (1.01%~6.66%), P4 (2.86%~6.65%), P5 (1.16%~5.43%), P7 (1.93%~5.46%), P11 (22.78%~46.33%), P12 (2.68%~31.17%), and P14 (1.01%~31.06%) were identified as the main components in the partial acid hydrolysates. Meanwhile, according to their relative retention times, peaks 2, 11, 12, and 14 were identified as Man, Glc, Gal, and Fuc, respectively. The other peaks might be oligosaccharides with different degrees and structures.

The HCA results of the partial acid hydrolysates are shown in Figure 4B, and the 12 batches of samples were classified into four categories based on common peak areas in the PMP–HPLC fingerprint. S2, S4, S7, and S9∼S11 with similar proportions of P2, P9, and P12 were clustered into category I, among which S9∼S11 and S7 had higher P5 proportions and were divided into two different groups. Category II contained S5 and S8, while only S12, with the lowest P12 proportion and highest P13 proportion, was grouped into category III. Three batches (S1, S3, S6) were clustered into category IV, and S6 with the highest P2 proportion was further separated from the other two samples. Moreover, the heatmap showed that P6, P11, P8, and P13 were the main components showing great differences among the strains, and components with different structures might affect the activity of the polysaccharides. The results also suggested that polysaccharides with similar monosaccharide compositions could be distinguished by analyzing the PMP–HPLC fingerprint.

### 2.4. Immune Activity Assay of Polysaccharides In Vitro

**Figure 5:**
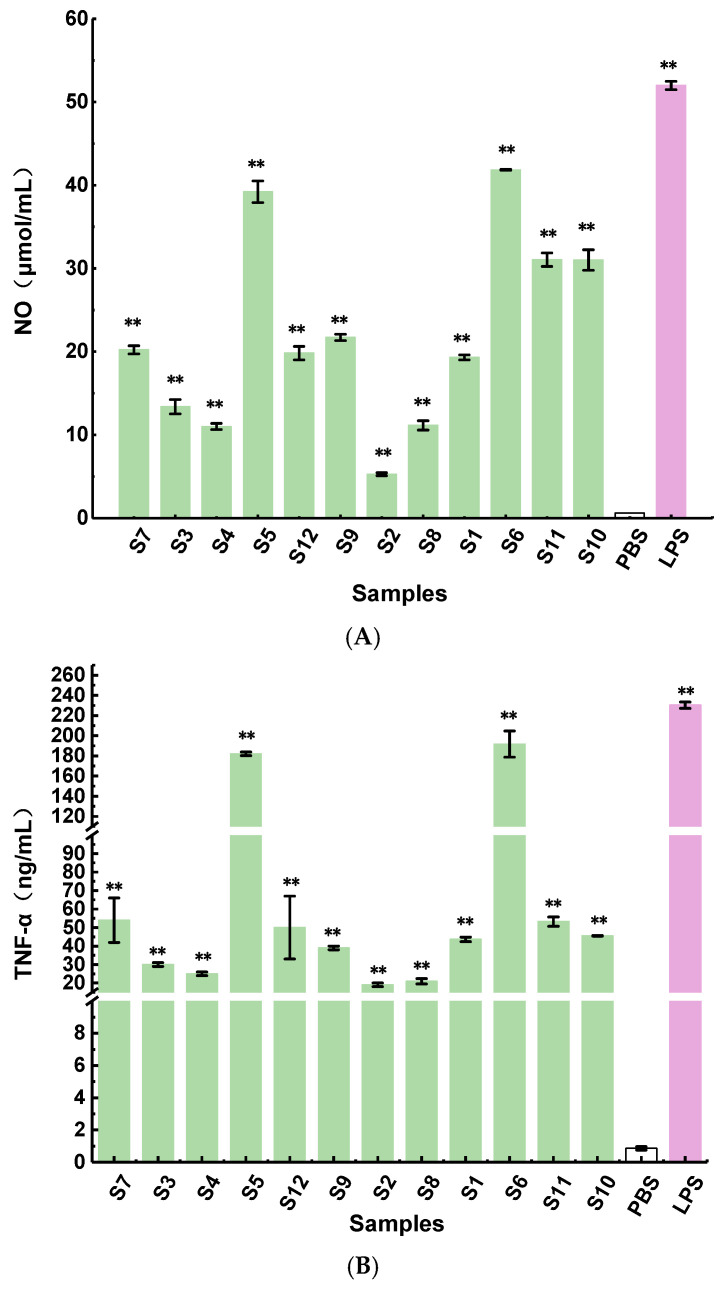
Effects of polysaccharide fractions on NO release (**A**) and TNF-α secretion (**B**) from RAW264.7 cells. ** indicates a significant difference between samples and negative control at *p* < 0.01.

It has been reported that heteroglycans and glucans exhibit immunomodulatory activities [37]. Nitric oxide (NO) is an important cytokine for the host to enhance the immune system, and the secretion of NO will increase when the macrophages are activated [38]. The results indicated that the polysaccharides from the fruit bodies of different *Ganoderma lucidum* strains could stimulate RAW264.7 macrophages to produce NO, but there were significant differences in NO production, ranging from 5 to 42 μmol/L at a concentration of 500 μg/mL (Figure 5A). Fractions S5 and S6 possessed higher immune activity than the others, while S2 showed the lowest macrophage activation activity with NO production of 5 μmol/L. TNF-α produced by macrophages is also an important immune factor that regulates the immune response [39]. It can be seen from Figure 5B that the fractions obtained from different *Ganoderma lucidum* strains could activate macrophages to release TNF-α. Moreover, compared with the blank control, the polysaccharides could significantly promote the production of TNF-α (*p* < 0.01). TNF-α secretion ranged from 19 to 179 ng/mL at the same concentration, and S5 and S6 exhibited the best immune activity, which was consistent with the results of NO release production. These results showed that polysaccharides could induce the secretion of NO and TNF-α and regulate the immune response. Moreover, most polysaccharides with strong immune activity had a high ratio of Man and Gal, which implied that the chemical features of the polysaccharides might affect immunomodulation. To elucidate the correlation between the structural features and immunomodulatory activity, the fingerprint–activity relationship was established by linear regression analysis methods.

### 2.5. The Fingerprint–Activity Relationship

#### 2.5.1. Molecular Weight and Immune Activity

Grey correlation degree analysis is a simple evaluation method for profile–effect relationships to obtain the main factors affecting bioactivity. Grey relational analysis is based on the geometric similarity of various factors in order to determine the correlations between factors. In general, efficacy was used as the reference sequence and the factors affecting bioactivity were used as the comparison sequences. Finally, the original data requires a series of calculations to obtain the correlation degree [40]. If the comparison sequence was a major factor influencing the reference sequence, then the variable had a high degree of correlation. In this study, the immune activity (NO production of RAW264.7) was set as the reference sequence ($x_{i}$) and the peak areas of 4 common peaks of the HPSEC fingerprint were used as the comparison sequences ($x_{i}$). The method cannot make direct comparisons between the comparison and reference sequences due to the different dimensions, thus the sequences were dimensionlessly processed according to Equation (1). Then, the correlation coefficient was calculated by Equation (2) according to a previous report [41], and the degree of correlation was obtained using Equation (3) to compare the overall information.

(1) $$x_{i}\left(k\right)=\frac{X_{i}\left(k\right)-X_{i}}{S_{i}}\;\left(i=0,\;1,\ldots ,\;n;k=1,\;2,\;\ldots ,\;m\right)$$

where $X_{i}\left(k\right)$: k_th_ factor in the ith sequence; Xi: average value of ith sequence; $S_{i}$: standard deviation of i_th_ sequence; and $x_{i}\left(k\right)$: dimensionless value of $X_{i}\left(k\right)$.

(2) $$\xi_{i}\left(k\right)=\frac{\begin{array}{c} min\\ i \end{array}\begin{array}{c} min\\ k \end{array}\begin{array}{c} \left|x_{0}\left(k\right)-x_{i}\left(k\right)\right|+ \end{array}\begin{array}{c} \rho max\\ i \end{array}\begin{array}{c} max\\ k \end{array}\begin{array}{c} \left|x_{0}\left(k\right)-x_{i}\left(k\right)\right| \end{array}}{\begin{array}{c} \left|x_{0}\left(k\right)-x_{i}\left(k\right)\right|+ \end{array}\begin{array}{c} \rho max\\ i \end{array}\begin{array}{c} max\\ k \end{array}\begin{array}{c} \left|x_{0}\left(k\right)-x_{i}\left(k\right)\right| \end{array}}$$

where $x_{0}\left(k\right)$: dimensionless value of the k_th_ factor of the reference sequence; ρ: a resolution coefficient ranging from 0 to 1, ρ = 0.5 usually used; and $\xi_{i}\left(k\right)$: grey correlation coefficient of the k_th_ factor in the *i*th sequence.

(3) $$\xi_{i}=\overset{m}{\underset{k=1}{\sum }}\frac{\xi_{i}\left(k\right)}{m}$$

where $\xi_{i}$: correlation degree of the *i*th comparison sequence.

The results indicated that the order of correlation degree was peak A2 (0.706) > peak A1 (0.654) > peak A3 (0.633) > peak A4 (0.560), suggesting that fraction A2 had the strongest correlation with immune activity of the polysaccharides, followed by A1, A3, and A4. Therefore, the A2 (4.27 × 10^3^~5.27 × 10^3^ g/mol) and A1 (1 × 10^4^~1.14 × 10^4^ g/mol) fractions, with high correlation degrees, might be the main bioactive components of the polysaccharides. Four polysaccharides (coded as CD1, CD2, CD3, CD4) with different molecular weights were isolated from six herbs, and the report indicated that CD3 with a higher molecular weight (1 × 10^4^~1 × 10^5^ g/mol) exhibited strong immunomodulatory activity [42] whereas CD4 (<1 × 10^4^ g/mol) exhibited weak immune activity, which was consistent with our study.

#### 2.5.2. Monosaccharide Composition and Immune Activity

Scatter plots can compare the relationship between two variables, which can be used as a preliminary analysis of variable correlation. The correlation between NO level and monosaccharide composition was analyzed by scatter plot in SPSS software. As shown in Figure S1, the monosaccharide content showed a certain correlation with the production of NO, and Gal, Man, and GluA were positively correlated with the NO level whereas Glc had almost no correlation with the NO level. Multiple linear regression analysis (MLRA) is used to analyze the relationship between a single dependent variable with two or more independent variables [43]. In this study, the MLRA model was used to explore the fingerprint–activity relationship based on the monosaccharide composition. The 7 common peak areas of the HPAEC fingerprint were extracted to establish the data matrices as independent variables (Table S1) and NO production from RAW264.7 cells was regarded as the dependent variable. The determination coefficient showed that the model could explain 72.2% of the variation. The mean value of the residuals was very close to zero (−4.07 × 10^−15^) and the R^2^ of the linear trend lines of the normal probability plots was 0.872, indicating that the residuals were normally distributed. These results demonstrated that the model fit well. According to the value of the standardized regression coefficient, the following regression equation could be obtained: Y = −0.208X_Fuc_ − 0.006X_Ara_ + 0.130X_GlcN_ + 0.604X_Gal_ − 0.395X_Glc_ + 0.415X_Man_ + 0.633X_GluA_. It was concluded that Gal, Man, and GluA with positive values had a positive correlation with immune activity whereas the content of Fuc and Glc displayed a negative correlation with immune activity. It was reported that polysaccharides isolated from *Flammulina velutipes* with a high ratio of Man and Gal possessed significant immune-enhancing activity by increasing the secretion of NO, TNF-α, IL-6, and IL-12 in macrophages, which was similar to our results [44]. Xiao et al. analyzed the correlation between monosaccharide composition and NO production for *Poria cocos* polysaccharides and found that the polysaccharides with high Gal and Man content had stronger immunological activity [45]. In addition, it was found that a heteropolysaccharide composed of glucose, galactose, mannose, and arabinose in the molar ratio of 8:5:4:1 could activate macrophages through their mannose receptor (MR), TLR4, and TLR2 [46]. However, another study reported that arabinose positively affected immunostimulation, which was different from our research [47]. This difference possibly may be related to the complicated structures of polysaccharides, since the activity of polysaccharides is affected by branching degree, linkage, conformation, etc. [48].

#### 2.5.3. Partial Acid Hydrolysates and Immune Activity

Grey correlation degree analysis was used first to analyze the influence of the components on immune activity. The immune activity (NO production of RAW264.7 cells) was set as the reference sequence and the peak areas of 14 common peaks in the PMP-HPLC fingerprint were used as the comparison sequences. The components with high correlation degree values contributed heavily to immune activity, and the results showed that peaks 2, 3, 4, and 8 had a high correlation with activity whereas peaks 5, 12, and 13 were weakly correlated with immune activity (Table 3). The above results indicated that the oligosaccharide hydrolysates were closely related to the immune activity of the polysaccharides and characteristic peaks related to activity were found. However, the positive or negative correlations between peaks and activity remained unclear and the fingerprint–activity relationship could not be quantified. Therefore, partial least-squares regression analysis was used to further explore the relationship between fingerprint and activity.

**Figure 6:**
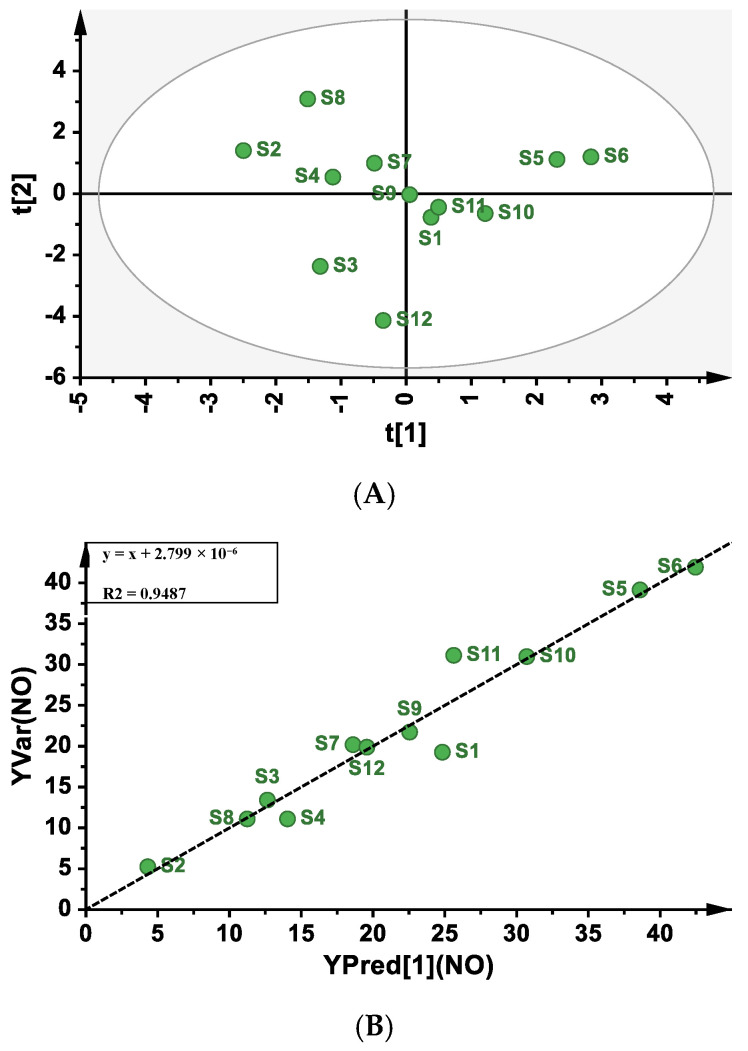

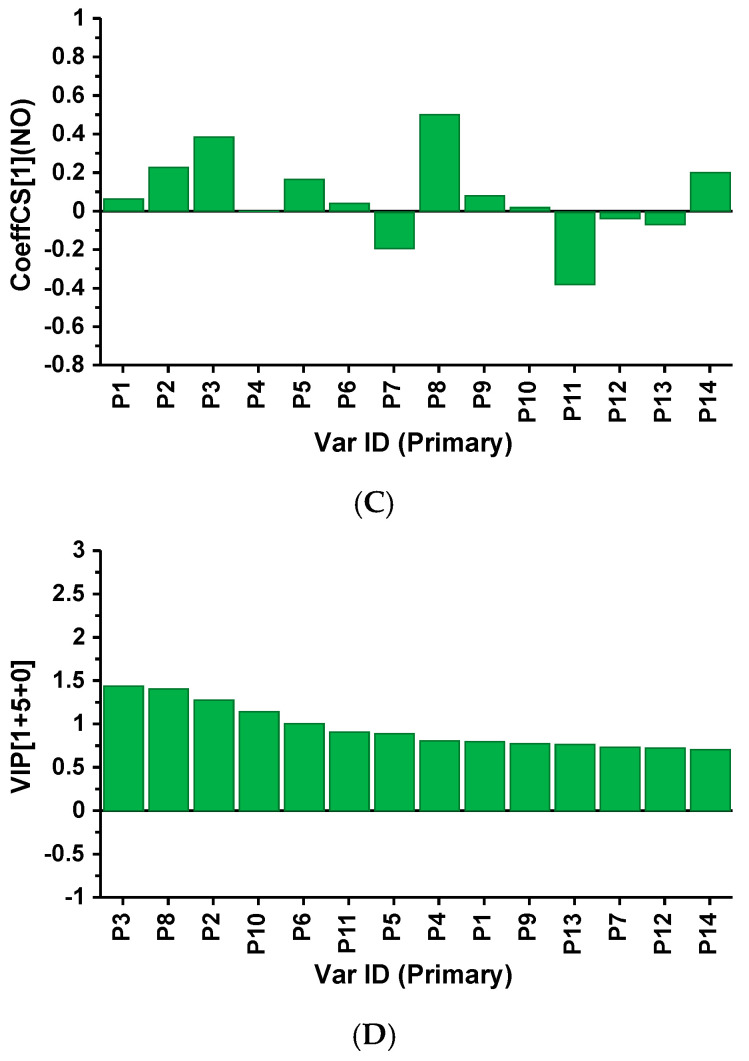
The correlation analysis of the PMP–HPLC fingerprint and immune activity through partial least-squares regression model. (**A**) The scatter plot scores of principle components; (**B**) The regression model between experimental and predicted NO values. (**C**) The coefficients plot; (**D**) The variable importance plot.

Partial least-squares regression analysis (PLSR) is a new multivariate statistical data analysis method that applies multiple linear regression analysis, canonical correlation analysis, and principal component analysis to process data at the same time. Moreover, the precision of PLSR model parameters improves with the increasing number of relevant variables and observations [49]. Therefore, PLSR is an ideal and powerful method to analyze the profile–effect relationship. The 14 common peak areas of the PMP-HPLC fingerprint were set as independent variable X (Table S2) and the NO production from RAW264.7 cells was set as dependent variable Y. The scatter plot scores of the principal component 1 (t1) and principal component 2 (t2) showed that S1-S12 were identified as normal samples as they were all distributed within the circle (Figure 6A). Thus, these samples could be used to establish the PLSR model. The linear equation between the predicted NO production obtained by the calibration model and the measured NO production was y = x + 2.799 × 10^−6^ (Figure 6B), the explanatory variance R^2^ of the model was 0.9487, and the predictive parameter Q^2^ was 0.717, which proved that the fitting and prediction ability of the model was good. From the coefficients plot (Figure 6C), it was found that P7, P11, P12, and P13 showed reversed columns, indicating a negative correlation between immune activity and fingerprint peaks, whereas the remaining 10 peaks had a positive correlation. In addition, the variable importance plot (Figure 6D) showed that P2, P3, P6, P8, and P10 had a significant effect on immune activity (VIP value > 1), which was partly consistent with the results of the grey relational analysis. The values of the correlation coefficients and VIP values of the 14 peaks with immune activity are shown in Tables S3 and S4. As a result, P2, P3, and P8 were speculated to be the main components possessing immune activity according to the comprehensive analysis. Furthermore, the results also provided direction for the subsequent purification and structural elucidation of active polysaccharides from *Ganoderma lucidum*.

In terms of the fingerprint–activity relationship, several factors including molecular weight, monosaccharide composition, and oligosaccharide fragments were associated with immune activity. In addition, it was found that polysaccharides with features such as higher *M_w_*, high proportions of Gal, Man, and GluA, and high proportions of P2, P3, and P8 exerted stronger immunomodulatory activity.

## 3. Materials and Methods

### 3.1. Materials

Twelve batches of *Ganoderma lucidum* (numbered S1 to S12) fruit bodies were collected from different places in China, including S1~S9 from Shouxiangu, Zhejiang Province, S10 from Huangshan, Anhui Province, and S11~S12 from Longquan, Zhejiang Province. The sample information is listed in Table 1. Trifluoroacetic acid (TFA), 1-phenyl-3-methyl-5-pyrazolone (PMP), monosaccharide standards (rhamnose, glucosamine, glucose, mannose, galactose, fucose, arabinose, fructose, xylose, glucuronic acid, and galacturonic acid), lipopolysaccharide (LPS), and polymyxin B were purchased from Sigma-Aldrich (St. Louis, MO, USA). Oligosaccharide standards including laminaribiose, laminaritriose, laminaritetraose, laminaripentaose, and laminarihexaose were purchased from Megazyme (Wicklow, Ireland). Pullulan standards P-5 (*M_w_* = 6300 g/mol) and P-10 (*M_w_* = 9800 g/mol) were purchased from Shodex (Tokyo, Japan). The RAW264.7 cell line was purchased from the Type Culture Collection of the Chinese Academy of Sciences. DMEM medium and fetal calf serum were bought from Gibco (Grand Island, NY, USA). The mouse TNF-α ELISA kit was bought from Beijing 4A Biotech Co., Ltd. (Beijing, China). All other reagents were analytical grade and produced in China.

### 3.2. Preparation of Polysaccharides

A total of 100.0 g of fruit bodies of *Ganoderma lucidum* was extracted twice with 2000 mL water for 2 h at 100 ℃, then the water extract was centrifuged (4 ℃, 4000× *g*, 15 min) and concentrated to 300 mL. Then, the extract was precipitated by adding ethanol to a final concentration of 50% to isolate the α/β-glucans. The supernatant was collected by centrifugation and precipitated using ethanol at a final concentration of 75%. The precipitate was collected, then washed twice with 75% ethanol, and dissolved in water to remove the residual ethanol. Finally, the solution of 75% ethanol precipitate was freeze-dried as the fraction with low *M_w_* for subsequent analysis. The yield of the fraction was expressed as (weight of the fraction/weight of the fruit bodies) × 100%.

### 3.3. Determination of Polysaccharide Content and Molecular Weight

The total content of polysaccharides was determined by the phenol–sulfuric acid method with D-glucose as a standard [50]. Molecular weights were determined using high-performance size exclusion chromatography (HPSEC) equipped with an eight-angle laser light scattering detector (MALLS, Wyatt Technology Co., Santa Barbara, CA, USA) and a refractive index detector (RI, Waters, Milford, MA, USA). TSK GEL G2500 PWXL and G3000 PWXL (7.8 mm × 300 mm, Tokyo, Japan) were used to separate the polysaccharide fractions. The molecular weights of the polysaccharides were calculated based on a standard curve derived from oligosaccharide and pullulan standards.

### 3.4. Fourier Transform Infrared Spectroscopy (FTIR) Analysis

A Nicolet is5 (Thermo Fisher Scientific, Waltham, MA, USA) was used to identify the functional groups of polysaccharides. Samples were mixed with KBr powder for FTIR analysis in the range of 4000~500 cm^−1^.

### 3.5. Monosaccharide Composition Analysis

Monosaccharide composition analysis was determined based on a previous report [51]. The sample (2 mg) was first hydrolyzed with 2 M TFA, and the hydrolysate was dried with nitrogen. Then, methanol was added to facilitate the volatilization of TFA, and the dried hydrolysate was dissolved with ultra-pure water. Finally, the solution was analyzed using high-performance anion exchange chromatography (HPAEC, ICS2500, Dionex, Sunnyvale, CA, USA) with a pulsed amperometric detector (PAD) and a Carbopac PA-20 column (3 mm × 150 mm, Dionex, Sunnyvale, CA, USA).

### 3.6. Partial Acid Hydrolysates Analysis

#### 3.6.1. Partial Acid Hydrolysis

The partial acid hydrolysis was based on a previous method with some modifications [52]. The polysaccharide sample (10 mg) was hydrolyzed with 1 mL of 1 M TFA in a small bottle at 80 °C for 3 h, and the solution was dried by blowing nitrogen after cooling to room temperature. Then, methanol (500 μL) was added to facilitate the volatilization of TFA, and this process was repeated four times.

#### 3.6.2. Preparation of PMP Derivatization and HPLC Analysis

PMP derivatization and HPLC were applied to determine the monosaccharides and oligosaccharides. The polysaccharide hydrolysates and monosaccharide standards were dissolved in water (100 μL), then 0.6 M NaOH (100 μL) and 0.5 M PMP–methanol solution (200 μL) were added, and the mixture was heated to 70 °C for 100 min. After cooling to room temperature, the mixture solution was neutralized with HCl and diluted with ultra-pure water. Finally, the derivatized product was extracted with chloroform (1 mL), and the organic phase was removed by centrifugation at 5000× *g* for 10 min. The extraction was repeated three times. The solution was passed through a 0.22 μm hydrophobic membrane for analysis [53].

The PMP derivatives were analyzed using the HPLC-DAD system (Waters, USA) equipped with a ZORBAX Eclipse XDB-C18 column (4.6 mm × 250 mm, Agilent Technologies, Santa Clara, CA, USA). The mobile phase was 0.1 M phosphate buffer (A) and acetonitrile (B) at a flow rate of 1 mL/min at 30 ℃. The gradient elution was as follows: 0~16 min, A/B (95/5→85/15, *v/v*); 17~60 min, A/B (83/17, *v/v*). The sample injection volume was 30 μL and the UV detection wavelength was set at 245 nm.

### 3.7. Immunomodulatory Activity Assay

#### 3.7.1. Cell Culture

RAW264.7 cells were cultured at 37 °C under 5% CO_2_ in DMEM supplemented with 10% heat-inactivated FBS (fetal bovine serum) and 1% penicillin–streptomycin. The RAW264.7 cells were resuspended to a density of 5 × 10^5^ cells per mL, and the cell suspension (180 μL) was added to 96-well culture plates. After incubation for 24 h, the polysaccharides (500 μg/mL) with polymyxin B (a specific inhibitor of LPS contamination) and LPS (10 μg/mL, lipopolysaccharides) were added to cells, respectively. Meanwhile, PBS (phosphate-buffered saline) was used as a blank control. After incubation for 48 h, the supernatants (100 µL) of each well were collected for the following test.

#### 3.7.2. Determination of NO and TNF-α In Vitro

The NO concentration of the cell supernatant was detected using the Griess method with NaNO_2_ as the standard [21]. The TNF-α secretion level was determined using a mouse TNF-α ELISA kit according to the manufacturer’s instructions.

### 3.8. Statistical Analysis

One-way analysis of variance (ANOVA) was used to identify differences between groups at *p* < 0.05 by SPSS 26.0 software. Microsoft excel software 2021 was used to analyze the grey correlation degree (GRA). Multiple linear regression (MLR) analysis on the fingerprint–activity relationship was performed using the enter method with SPSS 26.0 software [47]. Partial least-squares regression (PLSR) analysis on the fingerprint–activity relationship was performed using SIMCA-P 14.1 software.

## 4. Conclusions

In this study, 12 polysaccharides with low *M_w_* were isolated from different *Ganoderma lucidum* strains by ethanol precipitation and oligosaccharide fragments were prepared by acid degradation for further study. The results showed significant variations in the immunomodulatory activity of the polysaccharides among strains. The monosaccharide composition analysis indicated that the polysaccharides were mainly composed of Glc (54.16%~85.76%), Gal (4.88%~39.40%), and Man (0.26%~20.51%). Based on analysis of the fingerprint–effect relationship, it was revealed that fractions with *M_w_* of 4.27 × 10^3^~5.27 × 10^3^ g/mol were identified as the main bioactivity fractions, and high contents of Man, Gal, and GluA may further enhance the immune activity of polysaccharides as well as high contents of P2, P3, and P8 in the hydrolysate. These findings suggest that these components may play a crucial role in enhancing the immune activity of polysaccharides from *Ganoderma lucidum*, which has great significance for the quality evaluation of polysaccharides from *Ganoderma lucidum*. Moreover, it provides new insight into the fingerprint–activity relationship of polysaccharides. However, the fingerprints were insufficient to characterize the complete structures of the polysaccharides, thus more effort should put into the isolation and structural analysis of polysaccharides to explore the structure–activity relationship.

## Figures and Tables

**Table 1:** The yield and sugar content of the isolated polysaccharide fractions.

Sample No.	Yield/%	Sugar Content/%
S1	0.29 ± 0.01	46.37 ± 1.57
S2	0.34 ± 0.04	45.34 ± 3.30
S3	0.44 ± 0.03	39.59 ± 1.29
S4	0.22 ± 0.05	44.24 ± 2.45
S5	0.32 ± 0.02	46.41 ± 1.15
S6	0.20 ± 0.04	41.23 ± 1.6
S7	0.49 ± 0.04	40.80 ± 1.27
S8	0.74 ± 0.04	45.20 ± 1.27
S9	0.23 ± 0.03	41.30 ± 1.69
S10	0.18 ± 0.03	44.05 ± 1.39
S11	0.24 ± 0.03	48.96 ± 2.32
S12	0.37 ± 0.04	44.13 ± 2.69

**Table 2:** Monosaccharide composition and molar ratio of polysaccharides.

Sample No.	Monosaccharides (mol%)						
	Fucose	Arabinose	Glucosamine	Galactose	Glucose	Mannose	Glucuronic Acid
S1	1.77	0.18	1.99	11.82	75.26	7.05	1.40
S2	1.04	0.15	0.84	8.44	85.76	2.67	1.10
S3	2.87	0.25	1.77	4.88	78.25	8.42	0.97
S4	1.54	0.13	1.48	9.15	82.08	4.83	0.93
S5	1.74	0.45	4.2	16.01	54.16	20.51	1.65
S6	1.95	0.26	1.02	16.57	60.33	17.12	2.28
S7	0.82	0.30	2.11	14.43	69.58	11.18	1.58
S8	0.18	0.66	1.02	8.53	70.76	18.36	0.49
S9	1.08	0.12	0.86	15.09	74.22	6.70	2.04
S10	1.94	0.73	0.06	39.40	56.20	0.4	1.02
S11	1.75	0.27	1.34	20.87	73.77	0.26	1.54
S12	7.77	1.26	4.36	6.28	65.16	11.99	2.81

**Table 3:** The correlation degree of partial acid hydrolysates.

Peak No.	Correlation Degree	Peak No.	Correlation Degree
1	0.650	8	0.793
2	0.777	9	0.696
3	0.819	10	0.713
4	0.814	11	0.611
5	0.640	12	0.716
6	0.714	13	0.598
7	0.665	14	0.702

## Data Availability

The data presented in this study are available in the article and Supplementary Materials.

## Supplementary Materials

The following supporting information can be downloaded at: https://www.mdpi.com/article/10.3390/molecules28072913/s1, Table S1: Data matrix of the monosaccharide composition profile; Table S2: The percentage of common peak areas in the PMP–HPLC fingerprint; Table S3: The correlation coefficient of 14 common peaks based on PLSR; Table S4: The VIP values of 14 common peaks based on PLSR; Figure S1: The scatter plot of NO production and monosaccharides.

## Sample Availability

Samples of the polysaccharides are available from the authors.
