# Supplementary material for: Multiple Fingerprint–Activity Relationship Assessment of Immunomodulatory Polysaccharides from Ganoderma lucidum Based on Chemometric Methods
Source: Molecules. 2023 Mar 24;28(7):2913. doi: 10.3390/molecules28072913 (PMC10096448; doi:10.3390/molecules28072913)
Supplement: Supplementary file 1 [file molecules-28-02913-s001.zip › molecules-2224999-supplementary.pdf]

Supplementary data for

"Multiple fingerprint-activity relationship assessment of immunomodulatory polysaccharides from *Ganoderma lucidum* based on chemometrics methods"

Supplementary Figures

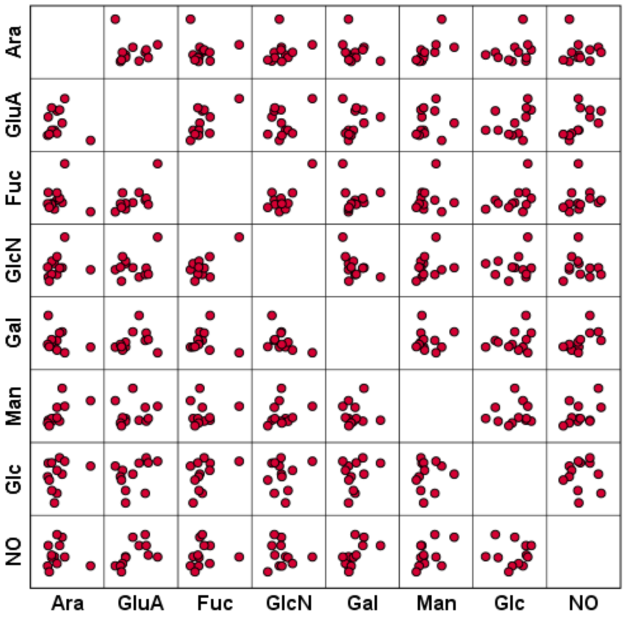

Figure S1. The scatter plot of NO production and monosaccharides.

Supplementary Tables

Table S1. Data matrix of the monosaccharide composition profile

Sample No.	Area of common peaks						
	1	2	3	4	5	6	7
S1	0.27	0.04	0.86	1.63	7.36	0.81	0.2
S2	0.16	0.03	0.37	1.19	8.39	0.46	0.16
S3	0.56	0.06	0.97	0.86	9.34	1.1	0.17
S4	0.26	0.024	0.71	1.41	8.72	0.68	0.15
S5	0.29	0.08	0.7	2.65	8.97	3.4	0.27
S6	0.36	0.06	0.53	1.8	7.09	1.9	0.39
S7	0.11	0.05	0.8	1.16	6.15	1.03	0.2
S8	0.04	0.19	0.65	1.15	9.76	2.43	0.1
S9	0.24	0.039	0.54	1.9	10.09	1.03	0.42
S10	0.57	0.026	0.47	4.25	10.05	0.89	0.33
S11	0.41	0.07	0.69	2.54	10.57	0.82	0.4
S12	1.36	0.09	1.45	0.6	10.23	1.99	0.51

1 to 7 represent the Fuc, Ara, GlcN, Gal, Glc, Man, GluA, respectively.

Table S2. The percentage of common peak area in PMP-HPLC fingerprint

Sample No.	Area of peak						
	P1	P2	P3	P4	P5	P6	P7
S1	1.14	6.28	1.86	4.55	3.03	0.28	4.61
S2	1.57	3.95	2.95	6.65	2.07	0.53	5.17
S3	0.89	2.74	1.41	4.65	1.72	0.56	3.03

S4	1.35	4.82	2.83	6.38	3.62	0.9	4.77
S5	0.85	10.12	5.07	2.86	1.16	4.91	2.35
S6	1.53	15.36	6.66	3.2	1.8	0.92	2.64
S7	0.66	6.61	1.61	4.37	7.69	0.58	2.58
S8	0.69	10.93	1.22	0.73	5.43	1.02	2.1
S9	1.25	6.12	2.06	5.62	3.07	0.62	3.55
S10	1.93	5.81	3.93	5.59	2.42	0.23	5.46
S11	1.5	6.13	2.42	5.32	2.73	0.3	5.43
S12	0.54	3.1	1.01	3.15	1.58	0.39	1.93

Continued table

Sample No.	Area of peak						
	P8	P9	P10	P11	P12	P13	P14
S1	1.46	0.26	2.73	34.41	19.06	4.71	15.63
S2	0.13	0.48	3.52	46.33	17.12	0.28	9.23
S3	0.17	0.4	4.75	32.57	6.8	17.48	22.85
S4	0.6	1.08	4.45	46.26	10.53	0.73	11.66
S5	0.8	1.37	1.93	25.77	31.17	2.23	9.43
S6	1.68	0.19	0.19	41.67	12.29	3.83	8.05
S7	0.45	0.5	4.55	36.96	26.84	0.95	5.65
S8	0.22	0.25	0.39	40.95	29.09	5.97	1.01
S9	1.14	0.6	3.5	38.23	20.67	0.65	12.93
S10	1.13	1.03	1.98	33.01	17.1	0.22	20.15
S11	1.69	0.49	2.6	39.8	15.1	0.26	16.23
S12	0.17	0.24	3.37	22.78	2.68	28	31.06

Table S3. The correlation coefficient of 14 common peaks based on PLSR

Peak No.	Correlation coefficient	Peak No.	Correlation coefficient
1	0.069	8	0.507
2	0.234	9	0.087
3	0.392	10	0.025
4	0.005	11	-0.381
5	0.171	12	-0.038
6	0.047	13	-0.071
7	-0.193	14	0.206

Table S4. The VIP values of 14 common peaks based on PLSR

Peak No.	VIP	Peak No.	VIP
1	0.809	8	1.419
2	1.289	9	0.790
3	1.451	10	1.158
4	0.821	11	0.923
5	0.902	12	0.735
6	1.02	13	0.777
7	0.746	14	0.719
